# Stress Conditions Induced by Carvacrol and Cinnamaldehyde on *Acinetobacter baumannii*

**DOI:** 10.3389/fmicb.2016.01133

**Published:** 2016-07-19

**Authors:** Angélique Montagu, Marie-Laure Joly-Guillou, Elisabeth Rossines, Jérome Cayon, Marie Kempf, Patrick Saulnier

**Affiliations:** ^1^LUNAM UniversitéAngers, France; ^2^INSERM U1066, Micro et Nanomédecines Biomimétiques, Institut de Biologie en Santé – Centre Hospitalier UniversitaireAngers, France; ^3^ATOMycA, INSERM Atip-Avenir Team, CRCNA, Inserm U892, 6299 CNRS, University of AngersAngers, France; ^4^Laboratoire de Bactériologie, Institut de Biologie en Santé – Centre Hospitalier UniversitaireAngers, France; ^5^Eydo PharmaChartres, France; ^6^Plateforme d’Analyse Cellulaire Et Moléculaire, Institut de Biologie en Santé – Centre Hospitalier UniversitaireAngers, France

**Keywords:** *Acinetobacter baumannii*, carvacrol, cinnamaldehyde, heat shock proteins, catalase, reactive oxygen species, defense mechanisms

## Abstract

*Acinetobacter baumannii* has emerged as a major cause of nosocomial infections. The ability of *A. baumannii* to display various resistance mechanisms against antibiotics has transformed it into a successful nosocomial pathogen. The limited number of antibiotics in development and the disengagement of the pharmaceutical industry have prompted the development of innovative strategies. One of these strategies is the use of essential oils, especially aromatic compounds that are potent antibacterial molecules. Among them, the combination of carvacrol and cinnamaldehyde has already demonstrated antibacterial efficacy against *A. baumannii*. The aim of this study was to determine the biological effects of these two compounds in *A. baumannii*, describing their effect on the rRNA and gene regulation under environmental stress conditions. Results demonstrated rRNA degradation by the carvacrol/cinnamaldehyde mixture, and this effect was due to carvacrol. Degradation was conserved after encapsulation of the mixture in lipid nanocapsules. Results showed an upregulation of the genes coding for heat shock proteins, such as *groES*, *groEL*, *dnaK*, *clpB*, and the catalase *katE*, after exposure to carvacrol/cinnamaldehyde mixture. The catalase was upregulated after carvacrol exposure wich is related to an oxidative stress. The combination of thiourea (hydroxyl radical scavenger) and carvacrol demonstrated a potent bactericidal effect. These results underline the development of defense strategies of the bacteria by synthesis of reactive oxygen species in response to environmental stress conditions, such as carvacrol.

## Introduction

Multi-drug-resistant (MDR) *Acinetobacter baumannii* isolates have emerged as a major worldwide cause of nosocomial infections, exhibiting ever-increasing rates of resistance (Perez et al., 2007). *A. baumannii* infections occur in intensive care units, where they are commonly found as the cause of ventilator-associated pneumonia, urinary tract infections, secondary meningitis, and bacteremia (Bergogne-Bérézin and Towner, 1996). Such strains have a remarkable ability to upregulate or acquire resistance determinants (Karageorgopoulos and Falagas, 2008). Thus, the combination of their wide range of resistant determinants and their environmental resilience transforms them into powerful nosocomial pathogens. The difficulty in treating infections due to MDR isolates has prompted the development of innovative strategies. Recently, the use of natural resources, especially essential oils (EOs), has been explored for prevention and treatment of MDR bacteria (Burt, 2004; Kon and Rai, 2012). Among them are carvacrol and cinnamaldehyde, which are major aromatic constituents (MACs) extracted from oregano and cinnamon EO, respectively. The cinnamaldehyde/carvacrol combination has already demonstrated antibacterial efficacy against a wide variety of microorganisms, including Gram-negative and Gram-positive bacteria (Didry et al., 1994; Michiels et al., 2007; Pei et al., 2009; Ye et al., 2013). However, the exact inactivation mechanisms of these MACs are not well known. The main action of these lipophilic compounds is likely due to structural changes in the bacteria. Thus, the hydroxyl functions (OH) of carvacrol facilitate the contact with the bacterial membrane and the penetration through the outer layers of the bacterial cell wall, causing disintegration of outer membrane components (Ultee et al., 2002; Di Pasqua et al., 2007; Palaniappan and Holley, 2010). This phenomenon induces morphological modifications of the bacterial membranes *via* the formation of pores, which results in ion and cellular component leakage and leads to bacterial death. Recently, a study demonstrated the crucial role of hydroxyl functions in the antibacterial activity of carvacrol after substitution of hydroxyl functional groups by fatty acids (Montagu et al., 2016).

Besides, it has been shown that cinnamaldehyde inhibits the amino acid decarboxylase activity of *Enterobacter aerogenes* (Wendakoon and Sakaguchi, 1995) and the bacterial cell division protein FtsZ in *Bacillus cereus* (Domadia et al., 2007). However, it is not able to disturb the outer cell membrane or deplete the intracellular ATP pool contray to carvacrol (Helander et al., 1998).

With the increasing interest in EOs, including MACs, as antibacterial compounds, a better understanding of the biological effects leading to cell death caused by MACs is needed for their application as antibacterial compounds.

Considering these challenges, we decided to investigate and elucidate the antibacterial action of carvacrol and cinnamaldehyde in *A. baumannii* at the molecular level.

The objective of this work was to study the inactivation of *A. baumannii* by carvacrol and cinnamaldehyde and to describe their biological effects on rRNA and genes involved in the response to environmental stress. The production of hydroxyl radicals following exposure to carvacrol was also studied to determinate its mechanisms of antibacterial activity.

## Materials and Methods

### Chemicals

5-Isopropyl-2 methylphenol (Carvacrol, Car) was purchased from Sigma–Aldrich (St. Louis, MO, USA). Trans-cinnamaldehyde (Cin) was purchased from Merck–Millipore (Molsheim, France). The lipophilic Labrafac^®^ WL1349 (caprylic/capric acid triglycerides) was purchased from Gattefosse S.A. (Saint-Priest, France). Lipoïd^®^ S75-3 (soybean lecithin at 69% of phosphatidylcholine) came from Lipoïd Gmbh (Ludwigshafen, Germany). Kolliphor^®^ HS15 (a mixture of free polyethylene glycol 660 and polyethylene glycol 660 (hydroxystearate)) was from BASF (Ludwigshafen, Germany) and NaCl was from Prolabo (Fontenay-sous-bois, France). Deionized water was acquired from a Milli-Q plus system (Millipore, Paris, France) and sterile water was from Cooper (Melun, France). Phosphate-buffered saline (PBS) was obtained from Lonza (Verviers, Belgium). Thiourea, a hydroxyl radical scavenger, and 2,2′-bipyridyl, an iron chelator, were obtained from Sigma–Aldrich.

### Preparation and Characterization of CarCin Lipid Nanocapsules

Lipid nanocapsules (LNCs) were prepared according to the method described by Montagu et al. (2016). Briefly, the formulation consisted of mixing all of the components, (Kolliphor^®^ HS15, Labrafac^®^ WL1349, Lipoid^®^ S75-3, NaCl, and deionized water) with magnetic stirring and heating from room temperature to 90°C. Two cycles of progressive cooling and heating between 90 and 60°C were then performed to homogenize the mixture. Then, active compounds were added to the mixture at different cycles according to their phase inversion temperature (PIT) when they are introduced in emulsion systems. Finally, an irreversible shock induced by a sudden dilution of the mixture with cold water (69.36% w/w) was performed according to the PIT of the final mixture. Slow magnetic stirring was then applied to the suspension for 5 min. The CarCin-LNCs (carvacrol and cinnamaldehyde loaded-LNCs) were obtained at a final concentration of 29 mg per g of LNCs suspension (17 mg/g of carvacrol and 12 mg/g of cinnamaldehyde). Blank-LNCs were prepared according to the method described by Heurtault et al. (2002).

The average hydrodynamic diameter and the polydispersity index (PdI) of the nanocapsules were determined at 25°C, in triplicate, using a Malvern Zetasizer Nano ZS (Malvern Instruments S.A., Worcestershire, UK). For the measurements, the LNC suspensions were diluted 1:60 (v/v) in deionized water.

### Bacterial Strain

In the study, we used *A. baumannii* (AYE-ATCCBAA-1710), a cephalosporinase-overproducing and expanded spectrum β-lactamase-producing (BLSE) strain resistant to most β-lactams, aminoglycosides, fluoroquinolones, chloramphenicol, tetracycline, and rifampin. This strain was involved in an outbreak in 54 healthcare facilities in France between April 2003 and May 2004 (Fournier et al., 2006).

### Determination of MICs

The antibacterial effects of Car, Cin, CarCin mixture, thiourea, and 2,2′-bipyridyl were evaluated by the minimal inhibitory concentrations (MICs) against *A. baumannii*. Thiourea in solid form was weighed and added to culture (stock solution of 1.8 M in BHI). Stock solution of 50 mM of 2,2′-bipyridyl was previously dissolved in ethanol (Merck). For the determination of the MICs, a bacterial suspension with a turbidity equivalent to a 0.5 McFarland standard was prepared (10^8^ CFU/ml) in broth liquid medium. The solution was diluted in brain heart infusion (BHI, bioMerieux, Marcy l’Etoile, France). Briefly, in each well of a 96-well plate, serially diluted compounds were used in the presence of bacterial suspensions. The control corresponded to bacteria without treatment. After a 24-h incubation at 37°C, the MIC values were determined as the lowest concentration of the antimicrobial compound that inhibited the visible growth of the microorganism tested.

### Time-Kill Assays

To determine the lethality of the different antibacterial compounds, time-kill studies were performed at the MIC of compounds in BHI with a bacterial suspension of 10^8^ CFU/ml. The bacterial counts were measured at 0, 1, 3, 6, and 24 h after incubation at 37°C. At these time points, aliquots of treated cells were harvested. Suitable dilutions were performed, and bacterial cells were plated on Columbia agar with sheep blood (GmbH, Wesel, Germany). After overnight incubation of the plates at 37°C, colonies were counted. All experiments were performed at least three times in independent conditions.

### Quantitative Real Time PCR

#### RNA Isolation and Characterization

After 0.5, 1, 2, 3, and 4 h of contact with Car, Cin, CarCin, CarCin-LNCs, and blank-LNCs at their MIC, treated bacterial cells were harvested, and washed in PBS with RNA Protect reagent. Total RNA was extracted and purified using a RNeasy Microkit (Qiagen, Courtaboeuf, France) and treated with DNase (10 U DNase I/μg total RNA). RNA concentrations were determined using a ND-2000 NanoDrop (Thermo Fisher Scientific, Wilmington, DE, USA).

RNA integrity and concentration were assessed with the Experion^TM^ automated electrophoresis station (Bio-Rad Laboratories Inc.) using the Experion RNA StdSens reagents and Experion RNA StdSens chips, according to kit instructions. The electropherograms and gels were evaluated using the Experion software version 3.0.

### cDNA Synthesis and Quantitative PCR

The values obtained for normalization of the RNA template were used in the reverse transcription reactions. First-strand cDNA synthesis was performed with SuperScript^TM^ II Reverse Transcriptase (Invitrogen), in combination with random hexamers, according to the manufacturer’s instructions. Following first-strand cDNA synthesis, cDNAs were purified (QIAquick PCR purification kit, Qiagen, Courtaboeuf, France) and eluted in 20 μL RNAse-free water (Gibco). Then, 1.25 ng of cDNA was mixed with the Maxima^TM^ SYBR Green qPCR master mix (Fermentas) and primer mix (0.3 μM) in a final volume of 10 μL. Targeted genes are presented in **Table 1**.

**Table 1:** Genes used in transcriptomic study.

Genes	Gene description	Functions	Reference
*ftsZ*	Filamantation temperature sensitive protein	Bacterial cell division	Domadia et al., 2007
*marR*	Multiple antibiotic resistance	Transcriptional regulator	Visvalingam et al., 2013
*yidC*	Membrane protein	Membrane protein translocation and insertion	Patil et al., 2013
*groES*	Chaperone HSP60	Protein refolding	Cardoso et al., 2010
*groEL*	Chaperone HSP60	Protein refolding	Cardoso et al., 2010
*dnaK*	Chaperone HSP70	Disaggregase activity	Cardoso et al., 2010
*katE*	Catalase	Detoxify reactive oxygen species	Imlay, 2003
*hsp15*	Heat shock protein (HSP)	Protein refolding	Cardoso et al., 2010
*ahpC*	Alkyl hydroperoxide reductase C22 subunit	Oxidative stress response	Benndorf et al., 2001
*ompA*	Outer membrane protein A	Porin protein	Visvalingam et al., 2013
*cfa*	Cyclopropane fatty acids	Membrane fluidity	Yoon et al., 2015
*clpB*	Chaperone HSP100	Disaggregase activity	Martin et al., 2014

These genes were *ftsZ*, *marR*, *yidC*, *groES*, *hsp15*, a*hpC*, *ompA*, *cfa*, *groEL*, *dnaK*, *katE*, and *clpB*, which are known to be involved in protective responses against stress in bacteria. Amplification was carried out on a LightCycler 480 (Roche) with a first denaturation step at 95°C for 10 min and 40 cycles of 95°C for 15 s, 60°C for 30 s. After amplification, a melting curve of the products determined the specificity of the primers for the targeted genes (**Table 1**). A mean cycle threshold value (Cq) was obtained from 2 measurements for each cDNA. Specific gene expression was calculated using the 2^-ΔΔCT^ method using *rec*A as calibrator. These studies were performed at least three times in independent conditions.

### ROS Quenching Experiments and Iron Chelator

To determine whether formation of ROS is involved in the mechanism of inactivation by carvacrol, a potent hydroxyl radical scavenger, thiourea and a iron chelator, 2,2′-bipyridyl were used. They were added to the culture simultaneously with carvacrol to demonstrate hydroxyl radical-mediated cell death. The concentrations used were below their MIC, that is, 34.5 mg/ml and 0.06 mg/ml for thiourea and 2,2′-bipyridyl, respectively. The concentration of carvacrol was chosen to reduce the initial cell population to more than 2 log_10_ cycles. The bacterial counts were measured at 0, 3, 6, and 24 h after incubation at 37°C. At the indicated time points, aliquots of treated cells were harvested, serial dilutions were performed and then bacterial cells were plated on Columbia agar containing sheep blood. After overnight incubation of the plates at 37°C, colonies were counted. All experiments were performed at least three times in independent conditions.

### Statistical Analysis

The data were obtained from at least three independent experiments. The results were expressed as the mean value ± SEM. Kruskal–Wallis ANOVA was performed with GraphPad PRISM, and differences were considered significant with a p value lower than 0.05.

## Results

### Physicochemical Properties of Unloaded LNCs and CarCin-LNCs

The diameter of the blank-LNCs was 48.3 ± 1.5 nm with a PdI lower than 0.2, indicating the monodispersity of the preparation. CarCin-LNCs had a diameter of 107.3 ± 0.6 nm, showing an increased size compared to blank-LNCs, depending on the concentrations of the compounds. Concurrently, an increase in the zeta potential (ZP) was observed for CarCin-LNCs compared to unloaded LNCs.

### Antibacterial Activity of Bioactive Compounds

The antibacterial activities of Car, Cin, CarCin, and CarCin-LNCs were evaluated by MIC. The time-kill assays of Car, Cin, and CarCin were studied with *A. baumannii*. Results showed that all of the MICs were in the range of 0.16 mg/ml to 0.31 mg/ml, except for the blank-LNCs, which had a MIC above 5 mg/ml (**Table 2**).

**Table 2:** MICs of Car, Cin, CarCin, CarCin-LNCs, and blank-LNCs against *Acinetobacter baumannii* [Car, carvacrol; Cin, cinnamaldehyde; CarCin, carvacrol/cinnamaldehyde (60–40%)].

	Carvacrol	Cinnamaldehyde	CarCin	CarCin-LNCs	Unloaded-LNCs
MIC (mg/ml)	0.16	0.31	0.16	0.31	≥5
					

Thiourea and 2,2′-bipyridyl had a MIC of 34.5 mg/ml (450 mM) and 0.06 mg/ml (390 μM), respectively, against *A. baumannii.*

**FIGURE 1:**
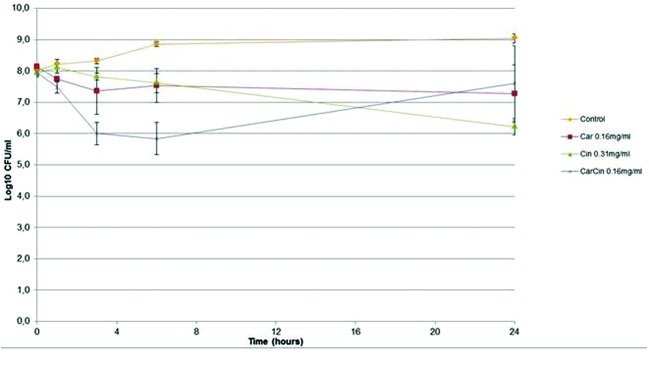
**Kill kinetics of Car, Cin, CarCin against *Acinetobacter baumannii* at the MIC [Car, carvacrol; Cin, cinnamaldehyde; CarCin, carvacrol/cinnamaldehyde (60–40%)] (data are expressed as the mean ± SEM)**.

For the kill kinetics, the concentrations used corresponded to the MICs for all antibacterials. A weak bactericidal effect (a difference of 2 logs copared to control) was observed for the compounds (**Figure 1**).

### Changes in Gene Expression after Exposure to Aromatic Compounds

**FIGURE 2:**
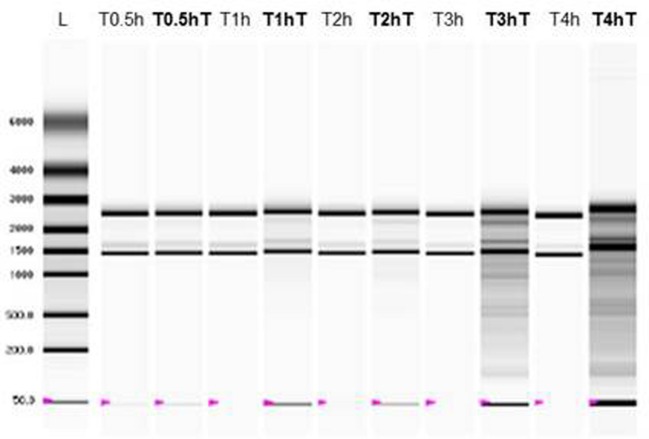
**RNA integrity of *A. baumannii* after CarCin exposure (CarCin, carvacrol/cinnamaldehyde (60–40%); T: with CarCin; L: ladder)**.

rRNA integrity of *A. baumannii* was evaluated after CarCin exposure at their MIC (**Figure 2**). The results demonstrated a modification of rRNA integrity after 3 h of CarCin exposure with a smeared band compared to the controls (two bands which corresponded to the 23S and 16S rRNA). To determine which compound(s) is (are) responsible for the rRNA degradation, Car and Cin were tested separately at the same time. After 30 min of exposure, results showed rRNA degradation induced by carvacrol but not by cinnamaldehyde (data not shown). No degradation of rRNA was detected for blank-LNCs, in contrast to CarCin-LNCs (data not shown).

**FIGURE 3:**
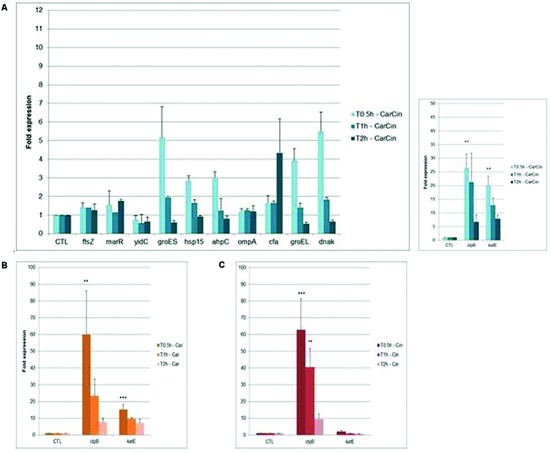
**Fold expression of genes after exposure of CarCin (A), Car (B), Cin (C) at 0.5, 1, 2 h in *A. baumannii* [Car, carvacrol; Cin, cinnamaldehyde; CarCin, carvacrol/cinnamaldehyde (60–40%)].** The data are representative of least three independent experiments. Points represent the means and bars represent the standard error of the mean of triplicate samples. ^∗∗∗^/^∗∗^/^∗^, *p* < 0.05.

Among the 12 genes studied, after 0.5 h of CarCin exposure, an overexpression between 3.9-fold and 5.1-fold of the *groES*, *groEL*, and *dna*K genes was observed (**Figure 3A**), followed by a decrease until normal levels at 2 h.

Besides, the genes encoding the heat shock protein (HSPs), *clp*B and the catalase *kat*E, were significantly upregulated at 0.5 h, by 26- and 20-fold, respectively, (*p* < 0,05). Later, the expression of these genes decreased slowly, respectively, from 21-fold and 12-fold at 1 h to sixfold and sevenfold at 2 h. No differential expression was observed with *fts*Z, *mar*R, *yid*C, and *omp*A after 0.5, 1, or 2 h of exposure. Finally, *cfa* gene was upregulated at 2 h (less then fourfold) (**Figure 3A**).

Treatment with Car or Cin alone for similar times induced an increase in the expression of *groES*, *groEL*, and *dnaK* genes at 0.5 h of exposure followed by a decrease to regain normal expression (data not shown). At 0.5 h, the highest expression was observed for the *clp*B gene, 60-fold in presence of Car and 62-fold with Cin. At 2 h, this expression decreased to sevenfold with Car and ninefold with Cin (**Figures 3B,C**). The *kat*E gene, which is known to be involved in oxidative stress response, was significantly upregulated at 0.5 h after Car exposure (15-fold) and decrease slowly at 2 h (sevenfold).

No differential expression was observed with blank-LNCs whereas overexpression of the *kat*E and *clp*B genes was observed after CarCin-LNCs exposure (^∗∗^*p* < 0.05 at 0.5 h for *clp*B) (data not shown).

### Involvement of Hydroxyl Radicals in *A. baumannii* Inactivation by Carvacrol ROS Quenching Experiments

To determine whether formation of reactive oxygen species (ROS) is involved in the mechanism of inactivation by carvacrol, a potent hydroxyl radical scavenger, thiourea, and a iron chelator, 2,2′-bipyridyl were added. Results showed that addition of thiourea and 2,2′-bipyridyl did not alter the growth kinetics, before 24 h (1– log_10_).

**FIGURE 4:**
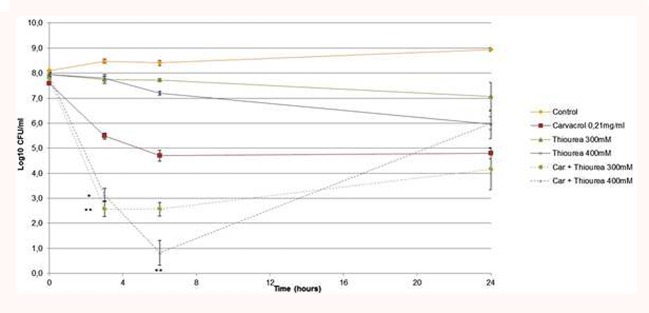
**Time kill assays of *A. baumannii* treated with carvacrol (0.21 mg/ml) in BHI broth at 37°C in the presence or absence of 300 mM or 400 mM thiourea (Car, carvacrol).** The data are representative of least three independent experiments. Points represent the means and bars represent the standard error of the mean of triplicate samples. ^∗∗^/^∗^, *p* < 0.05.

The kinetic assays showed that carvacrol had a bacteriostatic effect at 3 h and a bactericidal effect from 6 to 24 h (**Figure 4**). Evaluation of bacterial cells showed a bactericidal effect from 3 to 24 h when thiourea was added at 300 mM. Similarly, thiourea used at 400 mM with carvacrol had the same bactericidal profile until 24 h (^∗∗^*p* < 0.05). These results demonstrated a synergistic bactericidal effect between carvacrol and thiourea at the tested concentrations.

The results revealed that all combinations between 2,2′-bipyridyl and carvacrol had the same bacteriostatic kinetic profiles from 3 to 24 h (data not shown). These results indicated an improvement in bacterial cell survival after 2,2′-bipyridyl exposure.

## Discussion

The therapeutic challenges presented by *A. baumannii* infections are partially attributable to the fact that these bacteria exhibit significant genetic versatility in the acquisition of drug resistance and have the ability to survive in hospital environments (Bergogne-Bérézin and Towner, 1996; Peleg et al., 2008). The use of natural antimicrobials as alternatives to treat MRBs has gained popularity in recent years. The biological bases behind bacterial inactivation and resistance of carvacrol and cinnamaldehyde in *A. baumannii* have been little studied. The main action of these lipophilic compounds seems related to the direct interactions with the hydrophobic regions of the bacterial cell membrane (Burt, 2004).

In our study, we showed that the synergistic combination between Car and Cin was not relevant at the tested MICs. Nevertheless, to maximize the potential antibacterial activity, it was necessary to use a mixture of aromatic compounds acting on various antibacterial targets. Numerous studies have demonstrated the interest in combining these molecules on different species (Michiels et al., 2007; Santiesteban-López et al., 2007; Pei et al., 2009; García-García et al., 2011).

Moreover, the time-kill kinetics showed no bactericidal effect. This result is not surprising because this study has been performed with sublethal concentrations of antimicrobials and a high inoculum in order to maintain sufficient amounts of bacterial cells and to overcome the auto-lysis of bacterial cells for the genetic studies.

To evaluate the RNA integrity, we used ribosomal RNA (rRNA), composed of the 23S and 16S rRNA, and which constitutes more than 80% of total RNA (Clark and Pazdernik, 2012).

The results underlined an effect on rRNA only with Car, and not with Cin. These results are concordant with those from Helander et al. (1998). who showed that Cin is not able to penetrate thought the lipid bilayer, with those of Azirak and Rencuzogullari who demonstrated that Car and thymol significantly induced structural and total chromosome abnormalities in bone marrow cells of rats (Azirak and Rencuzogullari, 2008). Finally, the evaluation of rRNA integrity after exposure to encapsulated compounds showed earlier degradation of rRNA compared to CarCin. This result indicated the suitability of the carrier to promote a potential CarCin encapsulation. Furthermore, blank-LNCs demonstrated no rRNA degradation.

The transcriptomic response after 0.5 h of CarCin exposure showed upregulation of the *groES*, *groEL*, and *dnaK* genes. During stressful environmental conditions, such as the presence of aromatic compounds, bacterial pathogens generally increased their synthesis of HSPs and other stress proteins (Garbe, 1992). In bacteria, the major molecular chaperones include the dnaK and the groE machinery. These families of proteins are molecular chaperones or proteases. They play a part in facilitating refolding of damaged cytosolic proteins or in eliminating proteins that cannot be repaired (Mayhew et al., 1996).

We noted that the overexpression of HSP genes occurred immediately after exposure. After 2 h, bacteria established the normal expression of HSP genes. Many environmental triggers can induce HSP expression in bacteria, including alterations in the intracellular redox environment, exposure to heavy metals, amino acid analogs or cytotoxic drugs, glucose deprivation, and viral infection (Lindquist and Craig, 1988).

For example, a study demonstrated the induction of *dnaK* and *groEL* in response to antibiotics and HSP in *A. baumannii* (Cardoso et al., 2010). In our work, we demonstrated for the first time, the HSP gene induction by Car and Cin in *A. baumannii*.

Another HSP, *clpB* (HSP100), was significantly upregulated after 0.5 h CarCin, Car, and Cin exposure. The expression of *clpB* persisted over time, especially for Cin (*p* < 0.05 at 1 h), which was probably due to the biological effects of this compound. Indeed, this molecular chaperone collaborates with the HSP70 system in protein disaggregation, a crucial process for bacterial survival under stress conditions (Zolkiewski, 1999). Unfortunately, *clpB* is not well characterized, and its possible functional roles and potential protein substrates are unknown. However, it has been described as an essential factor to acquire thermotolerance and for the virulence of several Gram-negative (*Salmonella typhimurium* (Turner et al., 1998)) and Gram-positive bacteria [*S. aureus* (Frees et al., 2004), *Listeria monocytogenes* (Chastanet et al., 2004)].

In presence of Cin, we observed no differential expression of *ftsZ*, showing that Cin did not alter the *A. baumannii* morphology. A study conducted by Domadia et al. (2007) demonstrated that Cin at concentrations between 100 and 300 mg/L could also change the cell elongation in *E. coli* without substantial changes in viability (Visvalingam et al., 2013).

Late induction of *cfa* after 2 h of CarCin exposure was an adaptive response for modification of the membrane fluidity. It is known that under undesirable conditions, Gram-negative bacteria alter their membrane fluidity by regulating the ratio of unsaturated fatty acids (UFAs) to saturated fatty acids (SFAs). They also alter the levels of cfa or the conversion of cis to trans UFA (Yoon et al., 2015). The extent of cfa formation determines the fluidity of the membrane. Therefore, cfa formation increases as an adaptive response mechanism to several stresses, including low pH, salinity and the presence of toxic compounds, such as aromatic compounds, thereby enhancing the rigidity of membrane lipids (Denich et al., 2003). A study from Dubois-Brissonnet et al. (2011) showed an induction of fatty acid composition modifications by Car in a *Salmonella* strain Substantial changes were also observed in the long chain UFAs when the *E. coli* and *Salmonella* strains grew in the presence of Cin and Car, respectively (Di Pasqua et al., 2007).

Bacteria contain protective proteins that can detoxify ROS (sodA, sodB, sodC, AhpCF, KatG, KatE), establishing a defense mechanism against damage induced by ROS. In our case, only *kat*E gene was induced after Car exposure. The presence of hydrogen peroxide in the bacterial cells showed that Car induced oxidative stress. Free radicals or reactive oxygen intermediates are generated by cells during normal metabolism. When free radicals accumulate excessively, it leads to significant tissue damage and to a decrease in many cellular functions (Imlay, 2015). Car is able to damage rRNA at early exposure times through ROS production. However, Car is considered an antioxidant, and thus, it protects the cells against free radicals at sublethal concentrations (Guimarães et al., 2012; Ramamoorthy et al., 2014).

To determine whether Car acted on bacterial cells by ROS and assess the hypothesis that ROS contribute to antimicrobial lethality, a potent hydroxyl radical scavenger, thiourea, was used in combination with Car. This compound mitigated the effect of hydroxyl radical damage in bacteria (Repine et al., 1981; Touati et al., 1995). In our study, the combination of thiourea and Car induced a potent bactericidal effect and a recovery of bacterial growth after 6 h coinciding with thiourea elimination. We determined whether bacterial cells harvested at 24 h were resistant by performing a time-kill assay with the same treatment. The results demonstrated the same bactericidal profile. This result is not common because many studies showed the protection achieved by thiourea from bactericidal antibiotics (Kohanski et al., 2007), limonene (Chueca et al., 2014a), and Car and citral (Chueca et al., 2014b). In our case, we supposed that thiourea suppressed the defense mechanism of bacteria *via* ROS scavenging. Indeed, bacteria have developed some defense strategies involving ROS. ROS are also the response to environmental stress, such as Car. Therefore, bacteria could produce ROS to detoxify the bacterial environment by cleavage of OH function of carvacrol.

To determine the involvement of the Fenton reaction in the mechanisms of hydroxyl radical-mediated cell death, 2,2′-bipyridyl, an iron chelator that suppress the Fenton reaction, was added in combination with Car. The production of hydroxyl radical occurred following the Fenton reaction (reduction of hydrogen peroxide by ferrous ions). Nevertheless, adding the iron chelator to Car did not modify cell resistance to carvacrol. Thus, bacterial cells did not produce hydroxyl radicals via the Fenton reaction. However, we noted a slight bacterial activation, but it was not significant for all combinations at 24 h. Indeed, we demonstrated a bacteriostatic profile for all combinations tested contrary to Car alone (bactericidal profile). A similar result was obtained for citral with an iron chelator in *E. coli* (Chueca et al., 2014b).

## Conclusion

Our study demonstrated the biological effects of cinnamaldehyde and carvacrol on *A. baumannii* by a degradation of rRNA and an overexpression of genes encoding for HSPs and the catalase. The combination of thiourea and carvacrol showed a potent bactericidal effect underlying the development of defense strategies of bacteria using endogenous ROS in response to carvacrol.

## Author Contributions

AM made all experiences in the article. M-LJ-L and PS are the Ph.D. directors of AM. ER is a director of Eydopharma. JC formed AM about RT-qPCR experiences. MK corrected the article.

## Conflict of Interest Statement

The authors declare that the research was conducted in the absence of any commercial or financial relationships that could be construed as a potential conflict of interest.
